# Comprehensive chemical proteomics for target deconvolution of the redox active drug auranofin

**DOI:** 10.1016/j.redox.2020.101491

**Published:** 2020-03-03

**Authors:** Amir Ata Saei, Hjalmar Gullberg, Pierre Sabatier, Christian M. Beusch, Katarina Johansson, Bo Lundgren, Per I. Arvidsson, Elias S.J. Arnér, Roman A. Zubarev

**Affiliations:** a Division of Physiological Chemistry I, Department of Medical Biochemistry and Biophysics, Karolinska Institutet, 171 65, Stockholm, Sweden; b Science for Life Laboratory, Drug Discovery and Development Platform, Biochemical and Cellular Assay Facility, Stockholm, Sweden and Department of Biochemistry and Biophysics, Stockholm University, Stockholm, Sweden; c Division of Biochemistry, Department of Medical Biochemistry and Biophysics, Karolinska Institute, 171 65, Stockholm, Sweden; d Pfizer Innovations AB, 191 90, Sollentuna, Sweden; e Science for Life Laboratory Drug Discovery and Development Platform and Division of Translational Medicine and Chemical Biology, Department of Medical Biochemistry and Biophysics, Karolinska Institutet, 171 65, Stockholm, Sweden; f Sechenov First Moscow State Medical University, 119146, Moscow, Russia

**Keywords:** Ligand, Mechanism of action, Protein expression, Melting temperature, Target, CETSA, Cellular Thermal Shift Assay, FITEXP, Functional Identification of Target by Expression Proteomics, PISA, Proteome Integral Solubility Alteration assay, TPP, Thermal Proteome Profiling, TR-TPP, Temperature Range-Thermal Proteome Profiling, TXNRD1, Thioredoxin Reductase 1

## Abstract

Chemical proteomics encompasses novel drug target deconvolution methods in which compound modification is not required. Herein we use Thermal Proteome Profiling, Functional Identification of Target by Expression Proteomics and multiplexed redox proteomics for deconvolution of auranofin targets to aid elucidation of its mechanisms of action. Auranofin (Ridaura®) was approved for treatment of rheumatoid arthritis in 1985. Because several clinical trials are currently ongoing to repurpose auranofin for cancer therapy, comprehensive characterization of its targets and effects in cancer cells is important. Together, our chemical proteomics tools confirmed thioredoxin reductase 1 (TXNRD1, EC:1.8.1.9) as a main auranofin target, with perturbation of oxidoreductase pathways as the top mechanism of drug action. Additional indirect targets included NFKB2 and CHORDC1. Our comprehensive data can be used as a proteomic signature resource for further analyses of the effects of auranofin. Here we also assessed the orthogonality and complementarity of different chemical proteomics methods that can furnish invaluable mechanistic information and thus the approach can facilitate drug discovery efforts in general.

## Graphical abstract

**Figure:**
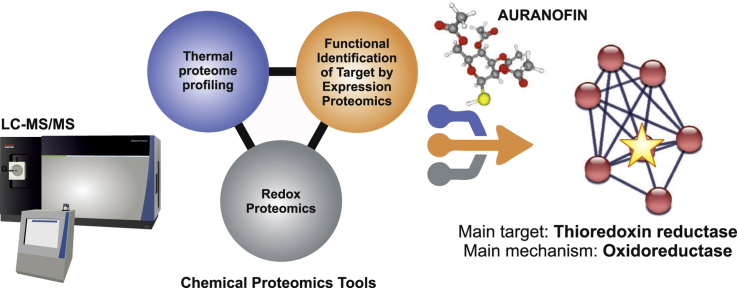

## 1 Introduction

Chemical proteomics has recently developed a set of tools for prediction of drug targets and determination of action mechanisms, in which drug modification is no longer required. These tools include Thermal Proteome Profiling (TPP) [1] and Functional Identification of Target by Expression Proteomics (FITExP) [2].

TPP is based on the well-established fact that ligand binding can change the thermal stability and/or solubility of a target protein [3]. Cellular Thermal Shift Assay (CETSA) [4] employed a Western blot format to probe such stability changes in lysate, living cells and tissues in a target-specific manner. Savitski et al. [1] extended CETSA to an untargeted, proteome-wide TPP assay where thousands of proteins are probed simultaneously after short incubation with the drug. TPP can be performed in temperature-range TPP (TR-TPP) format where protein stability is probed at biologically relevant concentrations in a range of temperatures [5]. In addition to identifying direct targets, TPP in cells can also reveal proteins which have altered stability downstream to any modulated activity of a direct drug target. TPP performed in lysate where most protein complexes are disrupted or weakened, can show proteins which directly interact with the compound [1]. Recently, we have greatly simplified the TPP-like workflow using a Proteome-wide Integral Solubility Alteration (PISA) assay [6].

In contrast to TPP, FITExP monitors changes in protein abundances after long-term (usually 48 h) incubation of living cells with the drug at a LC50 concentration. FITExP is based on the “the central dogma of expression proteomics”, which postulates that drug targets and main mechanistic proteins have the highest specific modulation of their expression levels upon a perturbation. FITExP has been used to predict targets and mechanisms of action of chemotherapeutics [2], metallodrugs [7], and nanoparticles [8]. Recently, we have shown that drug target deconvolution in FITExP can be improved by combining the proteomic data from treated matrix-detached (dying) and matrix-attached (surviving) cells [9]. We have further developed a tool called ProTargetMiner (http://protargetminer.genexplain.com), which is based on FITExP but employs a sizeable (>50 drugs) proteome signature library of anticancer compounds for highly specific target identification [10].

**Fig. 1:**
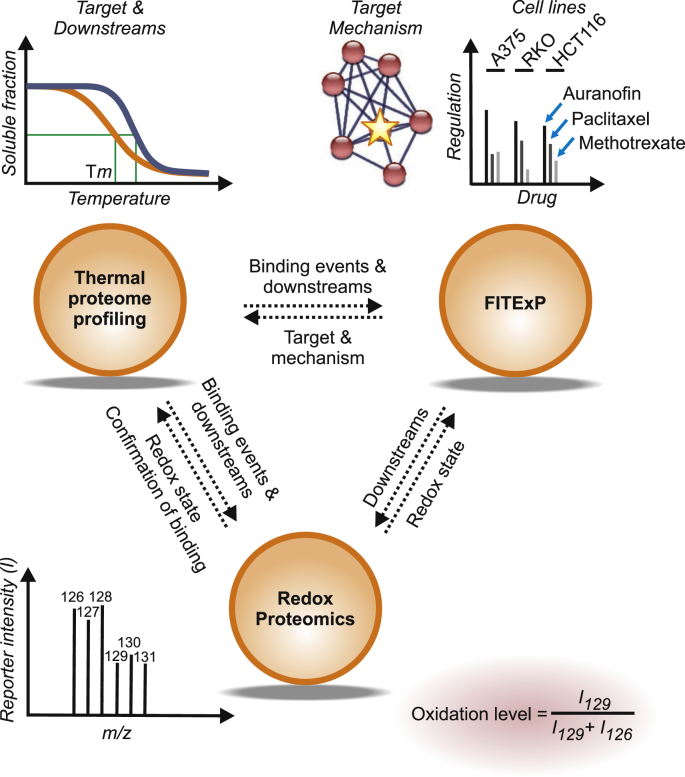
**Complementary chemical proteomics approaches for characterization of auranofin targets and mechanism space.** While TPP provides information related to stability changes in drug targets and downstream proteins, FITExP reveals proteins with affected abundances, which include both targets and mechanistic proteins. Redox proteomics reveals the changes in the oxidation states of cysteines at the peptide level, which can furthermore hypothetically correlate with protein stability changes in TPP.

Auranofin (Ridaura®) is a gold-containing molecule, approved by Food and Drug Administration (FDA) of the USA for treatment of rheumatoid arthritis. Given its potent antitumor activity [11], several clinical trials (ovarian cancer NCT03456700, chronic lymphocytic leukemia NCT03456700 and lung cancer NCT03456700) are underway, aiming to repurpose auranofin for cancer therapy [12,13]. Auranofin binds to its cognate selenoprotein target thioredoxin reductase 1 (TXNRD1) [14]. However, a controversy exists regarding cellular targets of auranofin, and several alternative action mechanisms have been proposed, some of which include inhibition of redox enzymes including primarily TXNRD1 [[15], [16], [17], [18]], inhibition of protein kinase C [19], inhibition of 3-hydroxy-3-methylglutaryl-coenzyme A reductase (HMGCR) [20], and protein tyrosine phosphatases [21]. Proteasomal deubiquitinase activities can also be inhibited, at least at higher concentrations of auranofin [15,22]. At the same time, potentially important off-targets of auranofin remain unexplored. To shed light on these issues, we decided to employ the above mentioned chemical proteomics approaches to characterize auranofin's cellular effects and deconvolute its targets in an unbiased *de novo* manner. Since auranofin is known to affect cellular redox balance, we also decided to add multiplexed redox proteomics to the chemical proteomics toolbox [23]. An overview of the three methods used and the complementary type of information gained from them is shown in Fig. 1.

## 2 Materials and methods

### 2.1 Cell culture

Human colorectal carcinoma HCT116 cells (ATCC, USA) were grown in McCoy's 5A modified medium (Sigma-Aldrich, USA) supplemented with 10% FBS superior (Biochrom, Berlin, Germany), 2 mM l-glutamine (Lonza, Wakersville, MD, USA) and 100 units/mL penicillin/streptomycin (Gibco, Invitrogen) and incubated at 37 °C in 5% CO2. Human skin malignant melanoma cells A375 and human colon carcinoma cells RKO were grown under the same conditions in DMEM. Cells were routinely checked for mycoplasma contamination by PCR.

### 2.2 Cell viability assay

Cell viability upon compound treatment was measured using CellTiter-Blue assay (Promega) according to manufacturer protocol and the LC50s were calculated, as the concentration of compound causing 50% cytotoxicity. The measured values have been listed in Supplementary Table 1.

### 2.3 TR-TPP experiment in lysate

The TPP experiment was performed according to Ref. [5] with some modifications. HCT116 cells were grown, trypsinized, washed and eventually lysed by 5X freeze-thawing in liquid nitrogen. The lysates were either incubated with 500 nM of auranofin or DMSO for 2 h. The lysate was then aliquoted into 10 microtubes each. The aliquots were incubated for 3 min at 37–67 °C (37, 41, 44, 47, 50, 53, 56, 59, 63 and 67) in SimpliAmp Thermal Cycler (Thermo). After heating, samples were kept at room temperature for 3 min and subsequently immediately snap-frozen in liquid nitrogen. The sample constituents were transferred into polycarbonate thickwall tubes and centrifuged at 100,000 g at 4 °C for 20 min using Optima LE-80 ultracentrifuge (Beckman). The soluble protein fraction was carefully transferred to new Eppendorf tubes. Protein concentration was measured in the samples treated with lowest temperatures (37 and 41 °C from each replicate) using Pierce™ BCA Protein Assay Kit (Thermo), the same volume corresponding to 50 μg of protein (in samples treated with lowest temperatures) was transferred from each sample to new tubes and sample buffer (8 M urea, 1% SDS, 50 mM Tris pH 8.5) was added. DTT was added to a final concentration of 10 mM and samples were incubated for 1 h at room temperature. Subsequently, iodoacetamide was added to a final concentration of 50 mM and samples were incubated in room temperature for 1 h in the dark. The reaction was quenched by adding an additional 10 mM of DTT. Proteins were precipitated using methanol/chloroform. After precipitation of proteins using methanol/chloroform, the semi-dry protein pellet was dissolved in 25 μL of 8 M urea in 20 mM EPPS (pH 8.5) and was then diluted with EPPS buffer to reduce urea concentration to 4 M. Lysyl Endopeptidase (Wako) was added at a 1 : 100 w/w ratio to protein and incubated at room temperature overnight. After diluting urea to 1 M, trypsin (Promega) was added at the ratio of 1 : 100 w/w and the samples were incubated for 6 h at room temperature. TMT10 reagents were added 4x by weight to each sample, followed by incubation for 2 h at room temperature. The reaction was quenched by addition of 0.5% hydroxylamine. Samples were combined, acidified by TFA, cleaned using Sep-Pak (Waters) and dried using a DNA 120 SpeedVac™ concentrator (Thermo). Samples were then resuspended in 0.1% TFA and separated into 8 fractions using High pH Reversed-Phase Peptide Fractionation Kit (Thermo). After resuspension in 0.1% FA (Fluka), each fraction was run with a 140 min gradient.

### 2.4 TR-TPP experiment in living cells

Cells were treated with 2 μM or 3 μM auranofin for 2 h inside the flasks, and were then trypsinized, washed twice with PBS, counted and distributed in 2 million cell aliquots inside PCR tubes. After heating, for cell lysis, samples were freeze thawed 3 times. The rest of the protocol was identical to the TR-TPP experiment in lysate.

### 2.5 Redox proteomics experiments

Cells were treated with 3 μM auranofin or corresponding amount of DMSO for 2 h, similar to the TR-TPP experiment. Fig. 4b shows the workflow of the redox experiment. Cell pellets were washed and lysed with 4 cell-pellet volumes of HES buffer (50 mM HEPES pH 8.0, 1 mM EDTA, 1% SDS and protease inhibitor). Samples were sonicated on ice for 45s, 30% amplitude, 3s on/off cycles using Branson probe sonicator. 50 μg protein was taken from each sample and incubated with the first set of 4.4 mmol/L iodoTMTX for 2 h at 37 °C with vortexing in the dark (free SH and SSH groups are labeled in this stage). Proteins were precipitated with methanol/chloroform to remove the excess iodoTMT. Samples were dissolved in HES buffer and incubated with 1 mM DTT for 1 h at 37 °C in the dark. Subsequently, 4.4 mmol/L of the second set of iodoTMT labels were added to the samples. Therefore, each sample was labeled with a distinct pair of labels to allow for pooling. The reaction was quenched by adding 20 mM final concentration of DTT and incubating samples for 15 min at 37 °C in the dark. After a second precipitation, protein pellets were dissolved in 100 μL of Tris and urea 8 M. Auranofin and DMSO-treated samples were pooled separately. After dilution of urea to 2 M, Lysyl Endopeptidase was added at a 1:100 w/w ratio overnight at room temperature. Urea was diluted to 1 M before trypsin addition for 6 h at 37 °C. Samples were acidified by adding TFA, cleaned using SepPak and lyophilized using a vacuum concentrator. The samples were fractionated into 8 using Pierce High pH Reversed-Phase Peptide Fractionation Kit (Thermo).

**Fig. 2:**
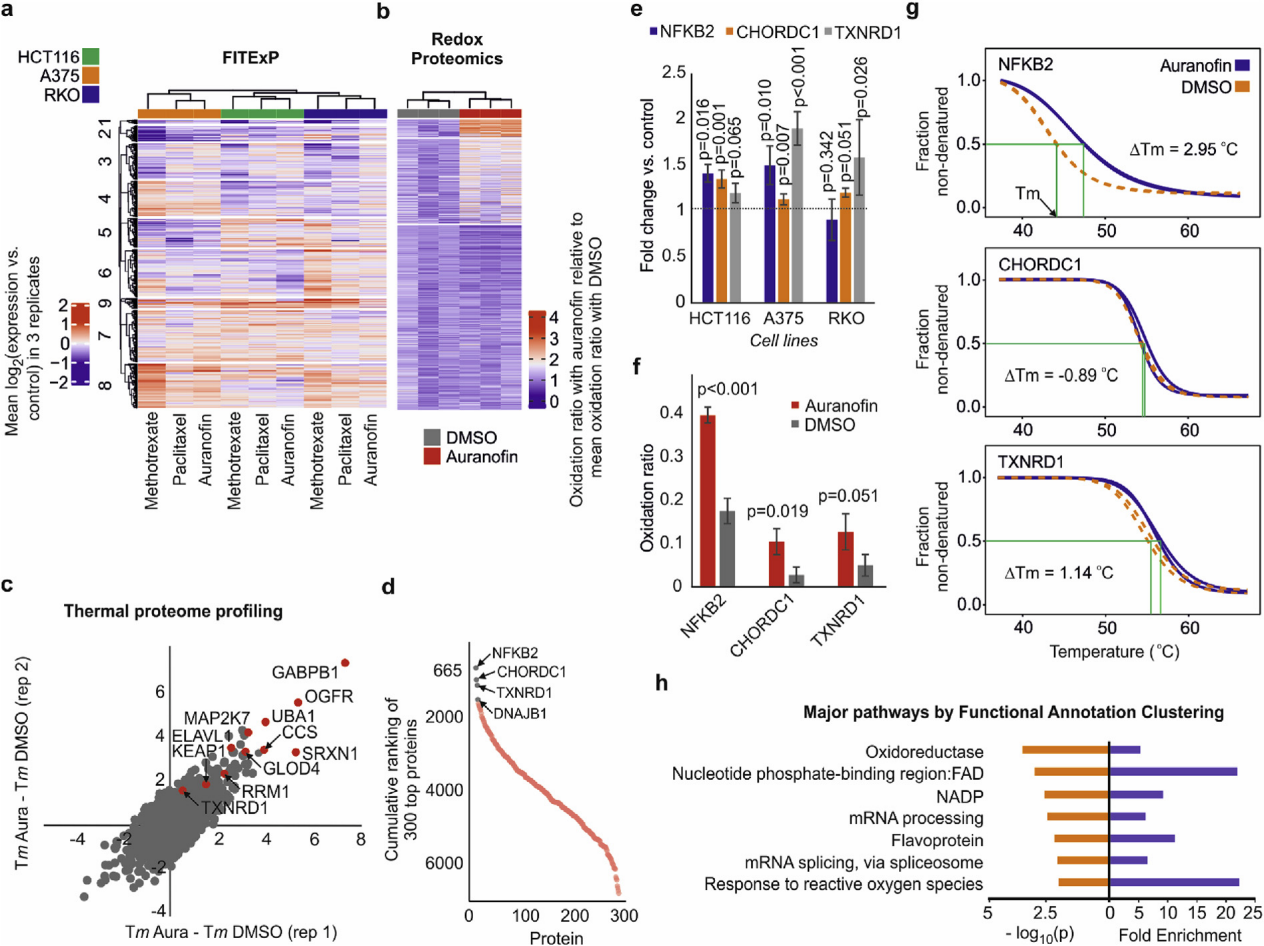
**Unbiased *de novo* prediction of auranofin targets and mechanism space by chemical proteomics tools. a**, Combining the FITExP data from three cell lines and three drugs, which was followed by identification of compound-specific proteome signatures by hierarchical clustering. Data in the heatmap is presented as mean log2 (fold change vs. control) in 3 replicates. b, Redox proteomics revealed an increase in the oxidation level of many peptide in response to auranofin, as expected. Data is plotted as oxidation ratio upon auranofin treatment relative to the mean oxidation ratio upon DMSO treatment. c, TPP data for proteins with ≤1 °C difference between the two replicates in 3 μM auranofin treatment of HCT116 cells shows stabilization of several proteins. Some of these proteins, including GABPB1 [30], RRM1 [31,32] and SRXN1 [33] are known to be regulated by the thioredoxin system. d, The cumulative sum of individual target rankings in four different types of analysis (FITExP, TR-TPP in cells and lysate, as well as deep redox proteomics). Proteins with the lowest overall sum are top candidate targets (TXNRD1 is the known cognate target). e-g, Changes in 3 top target proteins: (e) – specific expression in three cells lines in FITExP, (f) – oxidation level of top peptide (f), and (g) – melting curves in TPP experiments in cells (none of these proteins changed their stability in cell lysate). h, Top 15 proteins from each method were combined (60 proteins in total from 4 methods) and analyzed with Functional Annotation Clustering tool in DAVID. Top enriched biological pathways with minimal redundancy (fold enrichment > 5 and p < 0.01), representing the dominant mechanisms for auranofin, are shown (n = 3 biological replicates for all experiments, TPP experiment was performed in 2 replicates. *P* values were calculated with two-sided student t-test, mean ± SD).

**Fig. 3:**
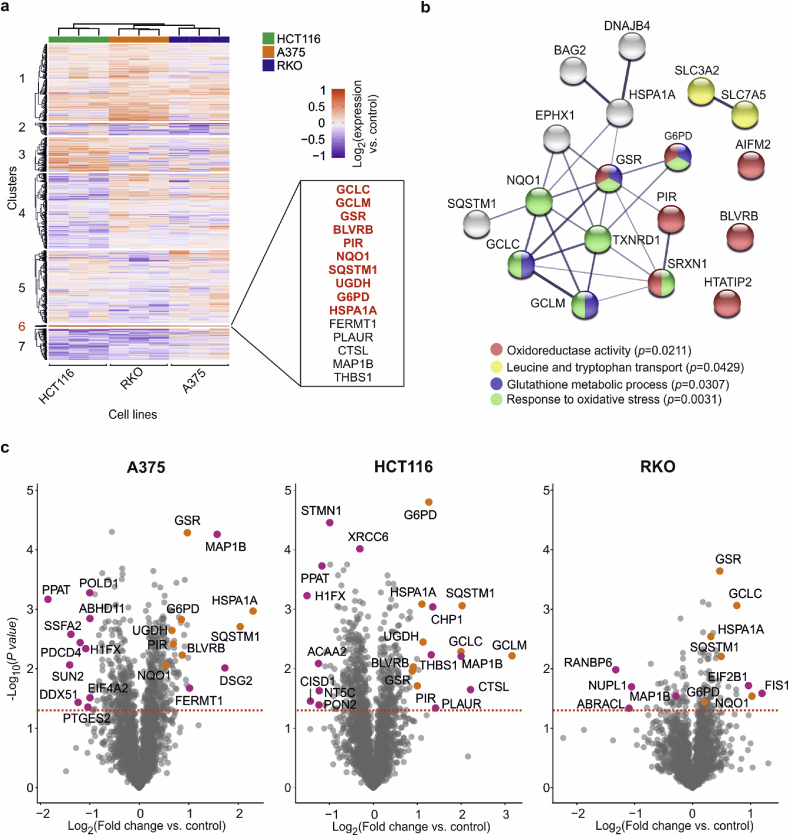
**Perturbation of “oxidoreductase” pathway as the dominant auranofin mechanism. a**, Clustering of FITExP data for auranofin in three cell lines in 3 replicates. Cluster 6 contains a group of tightly and consistently upregulated proteins. Most of these proteins are Nrf2 targets (bold red) [44,[51], [52], [53]]. The other 5 proteins might be putative Nrf2 targets. b, The enriched pathways for 30 most consistently upregulated proteins in three cell lines in FITExP (disconnected proteins have been removed). c, The consistent upregulation of Nrf2 target proteins from cluster 6 in (a) in different cell lines (in orange). Other significantly regulated proteins are shown in purple. (For interpretation of the references to color in this figure legend, the reader is referred to the Web version of this article.)

**Fig. 4:**
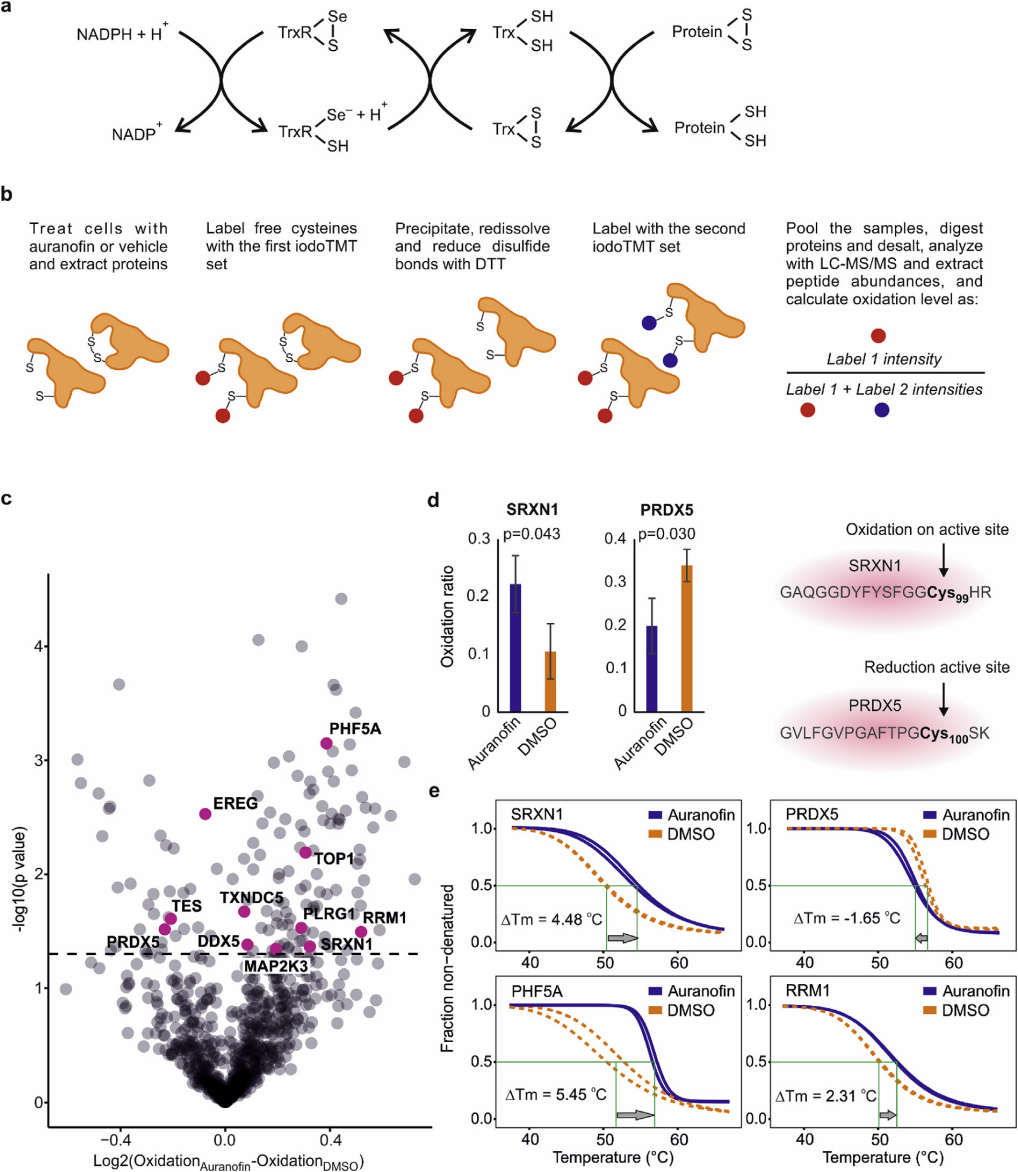
**Peptide oxidation state linked to protein stability. a**, Schematics of the role of TXNRD1 (TrxR) and the thioredoxin system in the cell and. b, redox experiment design with sequential multiplexed iodoTMT labeling to measure oxidation levels in the peptide level. c, Peptides from proteins with stability changes in TPP are highlighted in purple. d, Oxidation of SRXN1 and reduction of PRDX5 on the active sites. e, Change in the stability of SRXN1 and PRDX5 in opposite direction. Other representative proteins PHF5A and RRM1 had significantly oxidized peptides and were more stable upon exposure to auranofin. (*n* = 3 biological replicates for redox proteomics, TPP experiment was performed in 2 replicates. *P* values were calculated using two-tailed Student's t-test, mean ± SD). (For interpretation of the references to color in this figure legend, the reader is referred to the Web version of this article.)

### 2.6 FITExP experiment

For FITExP experiments, HCT116, A375 and RKO cells were treated with auranofin for 48 h at LC50 concentrations. Methotrexate and paclitaxel were included in these experiments to increase the specificity of detection for differentially expressed proteins. In brief, cells were cultured in 6 well plates at a density of 250 k per well, allowed to detach overnight and treated with the compounds at LC50 concentrations for 48 h. After the treatment period, cells were washed with PBS and lysed with 3% SDC in ambic buffer. Samples were sonicated for 45s, 30% amplitude, 3s on/off cycles using Branson probe sonicator. After total protein quantification using BCA assay, the protein amount in each sample was normalized. DTT was added to a final concentration of 5 mM and samples were incubated for 1 h at room temperature. Subsequently, iodoacetamide was added to a final concentration of 50 mM and samples were incubated in room temperature for 1 h in the dark. The reaction was quenched by adding an additional 10 mM of DTT. After dilution of SDC to 1.5%, digestion was performed at a ratio of 1:100 w/w overnight for Lysyl Endopeptidase. SDC was then diluted to 0.5% and trypsin was added at the same ratio for 6 h. Samples were acidified by TFA, cleaned using SepPak and lyophilized.

### 2.7 Proteomics

Proteins digests (1 μg) were analyzed in a randomized order by LC-MS/MS. Samples were loaded onto a 50 cm column (EASY-Spray, 75 μm internal diameter (ID), PepMap C18, 2 μm beads, 100 Å pore size) connected to an Easy-nLC 1000 pump (Thermo Fisher Scientific) with buffer A (0.1% formic acid in water) and eluted with a gradient reaching from 2% to 30% of buffer B (98% ACN, 0.1% FA, 2% H2O) at a flow rate of 250 nL/min. Details of all proteomic experiments have been summarized in Supplementary Table 2.

### 2.8 Data processing

The raw data from mass spectrometry were searched in MaxQuant, version 1.5.6.5, for quantification of proteins [24]. The Andromeda search engine [25] was run against the International Protein Index (human version UP000005640_9606, 92,957 entries). Methionine oxidation were selected as variable modifications, while cysteine carbamidomethylation was set as a fixed modification (expect for redox proteomics experiments where iodoTMT was chosen for quantification). No more than two missed cleavages were allowed and a 1% false discovery rate was used as a filter at both protein and peptide levels. All the contaminants were removed in the first step and only protein with at least two peptides were considered in all cases. Data were processed by Excel and R. The curve fitting was performed using the R package already made available in Ref. [5].

### 2.9 Ranking of target proteins in each experiment

The proteomics experiments were performed as follows: two TR-TPP in cells around auranofin LC50 concentrations (2 and 3 μM for 2 h in HCT116 cells) (data in Supplementary Tables 3 and 4, respectively), a TR-TPP in HCT116 cell lysate (500 nM for 1 h, Supplementary Table 5), a FITExP analysis with auranofin as well as methotrexate and paclitaxel as control compounds in RKO, HCT116 and A375 cells) (for each drug and cell lines, at a 48 h LC50 concentration, Supplementary Table 6), and a redox proteomics analysis under the same conditions as TR-TPP (3 μM auranofin for 2 h in HCT116 cells, Supplementary Table 7). The samples from redox proteomics were fractionated to increase the proteome coverage (Supplementary Table 8).

The ranking calculations for all experiments are provided in Supplementary Tables 4-6, 8. Data from TPP experiments with 3 μM auranofin in cells and 500 nM auranofin in lysate were used for the ranking, excluding proteins with missing abundance values and with >1 °C Tm differences between the replicates. The protein with the highest absolute mean Tm shift between the drug and vehicle treatments received rank 1, similar to other recent studies [26,27].

In the FITExP experiment that discovers the targets and mechanistic proteins consistently regulated by a drug in a panel of cell lines [10,28], only proteins quantified over the whole dataset were used in the ranking process. Proteins were ranked based on their mean regulation in each cell line; the most regulated protein received ranking 1. The individual rankings in three cell lines were summed together, with the sums sorted in the ascending order.

In the deep redox proteomics experiment, only peptides consistently quantified in all samples were used in ranking. Oxidation ratio was calculated as the abundance of peptides labeled with the second set of TMT tags (corresponding to peptides involved in disulfide bonds) normalized by the summed abundance of the first and second labels (all peptide population). As an output, the difference in the mean oxidation level of a peptide in auranofin and DMSO treatments was calculated. In addition, the *p* value was also calculated (two-tailed Student's t-test). The list of proteins was thereupon ranked twice: by the differences in oxidation ratios between drug and vehicle treatments (the highest difference received ranking 1), and by the p value. The two ranks were summed together, with the sums sorted in ascending order to obtain the final rank (Supplementary Table 8).

For each protein, all the above individual rankings were summed and sorted in ascending order to obtain the overall ranks (Fig. 2d and Supplementary Table 9). In redox proteomics, where more than one peptide could sometimes be quantified for each protein, the peptide with the best ranking was used in calculation of the cumulative rank, while other peptides from that protein were ignored.

### 2.10 Network mapping

The differentially regulated proteins were projected to STRING v10.5 (http://string-db.org) to map the protein-protein interaction networks and enrich for biological pathways. Medium confidence threshold (0.4) was used to define protein-protein interactions.

### 2.11 Statistics

The TPP experiments were performed in 2 replicates. All other experiments were performed in 3 independent replicates. Two-tailed t-tests were used throughout the paper for analysis of significance.

### 2.12 Data availability

The LC-MS/MS raw data files and extracted peptides and protein abundances are deposited in the jPOST repository of the ProteomeXchange Consortium under the dataset identifier PXD016776 [29].

## 3 Results and discussion

### 3.1 Unbiased prediction of auranofin targets using a combination of chemical proteomics tools

Since each chemical proteomics method yields both false positives and false negatives, parallel tools must be employed to obtain a reliable list of target protein candidates, each supported by, preferably, more than one technique. Thus, a series of proteomics experiments were performed, as detailed in Materials and Methods. Fig. 2a gives an overview of the data obtained in the FITExP experiment. Each column in the heatmap represents the mean log2 (fold change vs. control) in 3 replicates. Fig. 2b shows the overall results of the redox experiment, where data is plotted as oxidation ratio upon auranofin treatment relative to the mean oxidation ratio upon DMSO treatment. The heatmap shows an increase in the oxidation level of many peptides in response to auranofin, as expected. In Fig. 2c, we plot the TPP results in cells treated with 3 μM auranofin. Only the proteins with a confident Tm (within ±1 °C difference between the replicates) were considered.

To identify the most probable target candidates, the proteins were ranked in TPP based on the melting temperature difference (ΔTm) between auranofin and control treatments. In FITExP, the ranking was based on the absolute magnitude of consistent regulation in three cell lines, and in redox proteomics, based on the differences in the oxidation levels between the auranofin and control treatments (the top protein in each method got ranking 1). The rankings from all four experiments (TPP in cells and lysate, FITExP and deep redox proteomics) were summed up to shortlist the candidate drug targets (Fig. 2d) (proteins with the lowest overall rankings are top candidate targets; the top proteins are given in Supplementary Table 10). Although combining orthogonal chemical proteomics tools is a powerful approach in target deconvolution, one limitation is that not all proteins are quantified in all experiments (so-called missing value problem). Therefore, proteins quantified in all approaches are more likely to receive better rankings. This shortcoming will be ameliorated in the future by higher coverage of the proteome brought about by advances in mass spectrometry based proteomics.

TXNRD1 was found on the 3rd position. That the auranofin cognate target appeared among the top candidates testifies to the correctness of the performed chemical proteomics analyses, and independently validates TXNRD1 as a main auranofin target in cells. The fact highlighting the importance of combining several methods is that the highest ranking TXNRD1 received in a single analysis method was the 12th position in FITExP. The upregulation of TXNRD1 was cell-line dependent (Fig. 2e). The top detected oxidized peptide of TXNRD1 was CDYENVPTTVFTPLEYGACGLSEEK, with an auranofin to DMSO oxidation ratio of 2.53 (p = 0.051) (Fig. 2f). TXNRD1 was only slightly (but reproducibly) stabilized in intact cells (1.14 °C with 3 μM and 1.02 °C with 2 μM auranofin) (Fig. 2g), but not in cell lysates. The weak stability/solubility change of TXNRD1 may be attributed to the fact that auranofin binds to its penultimate amino acid (selenocysteine), with possible transfer of gold to redox-active cysteine couples inside the protein occurring next [34]. The near-terminus binding may not induce significant change in the overall thermal stability or solubility of the molecule. The C-terminal active site motif of TXNRD1 known to bind auranofin, contains a selenocysteine that is apparently not amenable to iodoTMT labeling as we did not detect the corresponding peptide.

The top protein was NFKB2 (Nuclear factor NF-kappa-B p100 subunit). Inhibition of NF-kB activation is known to be partially responsible for the anti-inflammatory effects of auranofin [35,36], and, interestingly, NF-kB signaling is also regulated by TXNRD1 and the thioredoxin system [[37], [38], [39]]. Here we found that NFKB2 was upregulated by auranofin about 1.4- and 1.5-fold in HCT116 and A375 cells, respectively, but slightly down-regulated to 0.9 in RKO cells (Fig. 2e). The oxidation of the NFKB2 top peptide QCSELGICAVSVGPK in the presence of auranofin was 2.3 times higher than for DMSO (p < 0.001) (Fig. 2f). Furthermore, NF-kB2 was stabilized by 2.95 °C in cells treated with 3 μM auranofin (Fig. 2g), but no significant stabilization was found in lysate.

CHORDC1 (Cysteine and histidine-rich domain-containing protein 1) that ranked second was unexpected among the top proteins. Despite being rich in cysteine, it appears to be a novel player in the context of thiol reactive metal compounds such as auranofin, and is mostly known for being involved in HSP90 chaperone complex and stress response [40]. CHORDC1 was upregulated in HCT116 and RKO cells by 1.4- and 1.2-fold, respectively, but its expression change was insignificant in A375 cells (Fig. 2e). The cysteine in the top peptide TSDFNTFLAQEGCTK (Cys211) was oxidized in the presence of auranofin 3.8-fold more than in DMSO (p = 0.019) (Fig. 2f). In TPP, CHORDC1 showed no stabilization in either cells (Fig. 2g) or lysate. Therefore, the appearance of CHORDC1 on the top of the list is mainly due to its significant oxidation shown in redox proteomics.

### 3.2 Pathway analysis reveals major auranofin mechanism

To reduce the risk of false negatives arising due to taking an interception of top results from different analyses, fifteen top proteins from each of the four methods were combined and subjected to pathway analysis by Functional Annotation Clustering tool in DAVID, where the number of redundant biological pathways is reduced by grouping similar annotations together [41]. The enrichment results are shown in Fig. 2h, and “oxidoreductase” is the top pathway, in line with available literature for auranofin.

To understand the cellular pathways perturbed by auranofin, the expression data for auranofin were clustered (Fig. 3a). Expectedly, the samples first clustered according to the three cell lines and then to the treatments. The protein Cluster 6 was composed of 15 proteins which were upregulated in all three cell lines, and which grouped together regardless of the chosen number of clusters (*n* = 6–10). This set of proteins best mapped to “glutathione metabolic process” (4 proteins, *p* < 0.0004) and “response to oxidative stress” (4 proteins, *p* < 0.0005), ranked by *p* values in StringDB (all pathways listed in Supplementary Table 11). Furthermore, pathway analysis of 30 top consistently upregulated proteins in the panel of cell lines in FITExP indicated the significant enrichment of the “oxidoreductase activity” (details in Supplementary Table 12) (Fig. 3b). The 30 most consistently down-regulated proteins in the panel of cell lines mapped to “nucleosome” and “histone H5” (details in Supplementary Table 13).

Despite differences in proteome responses between different cell lines, the upregulation of proteins involved in glutathione metabolism was observed in all cell lines (Fig. 3c). The upregulation of enzymes involved in glutathione metabolism is in line with glutathione being a backup system for TXNRD1 under oxidative stress conditions [42]. Interestingly, most of the consistently upregulated proteins are Nrf2 target (inducible) genes (bold red in Fig. 3a). Therefore, the other proteins in this list might also be Nrf2 inducible proteins. Inhibition of TXNRD1 typically leads to Nrf2 activation [43], which in turn activates genes involved in the response to oxidative stress such as GCLM and GCLC, which are involved in glutathione (GSH) synthesis [44], among others (Nrf2 inducible proteins upregulated in each cell line are shown in Fig. 3c). In cells treated with auranofin, Nrf2 is strongly activated [45,46]. As shown in Fig. 2c, KEAP1, the other member of the Keap1-Nrf2 cytoprotective pathway was also stabilized by 1.7 °C, but only in cells [47]. The pharmacological activity of auranofin has been shown to be associated with induction of heme oxygenase 1 (HMOX1) [48], which is also an Nrf2-activated gene [49] and upregulated upon TXNRD1 targeting [50]. Indeed, we noticed a 3.6 fold increase in HMOX1 levels in RKO cells, and >159-fold increase in HCT116 cells. Similarly, HMOX1 levels were almost undetectable in A375 cells before treatment (it was only detected in 1 of the replicates, despite the fact that the protein was identified with 22 unique peptides in other samples), but HMOX1 was reliably quantified after treatment with high intensity, meaning its significant induction. Since HMOX1 has cytoprotective effects, the severe induction of HMOX1 by auranofin might explain why auranofin demonstrates synergistic lethality with HMOX1 inhibitors against chronic lymphocytic leukemia (CLL) [49,50].

### 3.3 Cysteine oxidation in redox proteomics can be linked to protein stability in TPP

The thioredoxin system is responsible for reduction of other cellular proteins by cysteine dithiol-disulfide exchange (schematics in Fig. 4a). Therefore, inhibition of this system by auranofin could lead to stabilization of some redox-sensitive cellular proteins due to structural modulation [27,54]. To be able to compare the changes in the proteome redox state with the thermal stability changes, cells were treated at the same conditions as in TPP over 2 h with 3 μM of auranofin and then subjected to sequential iodoTMT labeling (Fig. 4b). This method will also detect persulfidated cysteine residues [55]. Of the 4459 peptides quantified, 869 had no missing values and were used in subsequent analyses. The overall oxidation level was higher for auranofin than DMSO, as expected, which resulted in an asymmetric volcano plot in Fig. 4c. At least 11 significantly oxidized or reduced peptides belonged to proteins with increased or decreased thermal stability in TPP, respectively (Supplementary Table 14).

The active site of SRXN1, Cys99 (the only cysteine residue in this protein) [56] was more oxidized (auranofin/DMSO ratio of 2) (Fig. 4d) and the protein was stabilized by +4.5 °C in TPP (Fig. 4e). Similarly, the active site of PRDX5, Cys100 [57,58] was less oxidized upon treatment with auranofin (auranofin/DMSO ratio of 0.6) (Fig. 4d), and the protein was also less stable in TPP (by −1.7 °C) (Fig. 4e). Other representative proteins with similar behavior are shown in Fig. 4e. Therefore, the combination of TPP and redox proteomics can more reliably identify potential redox regulatory switches in proteins than either of these methods separately.

The 105 significantly oxidized peptides in the redox experiment mapped best to the following KEGG pathways: ribosome, spliceosome, glycolysis/gluconeogenesis, metabolic pathways, DNA replication, cell cycle and protein processing in endoplasmic reticulum (the number of pathways is extensive, and the list of proteins can be found in Supplementary Table 7). The 35 significantly reduced peptides mapped most significantly to focal adhesion, adherens junction, ribonucleoprotein complex and proteasome regulatory particle (the list of proteins can be found in Supplementary Table 7).

### 3.4 TPP and FITExP are mostly orthogonal

**Fig. 5:**
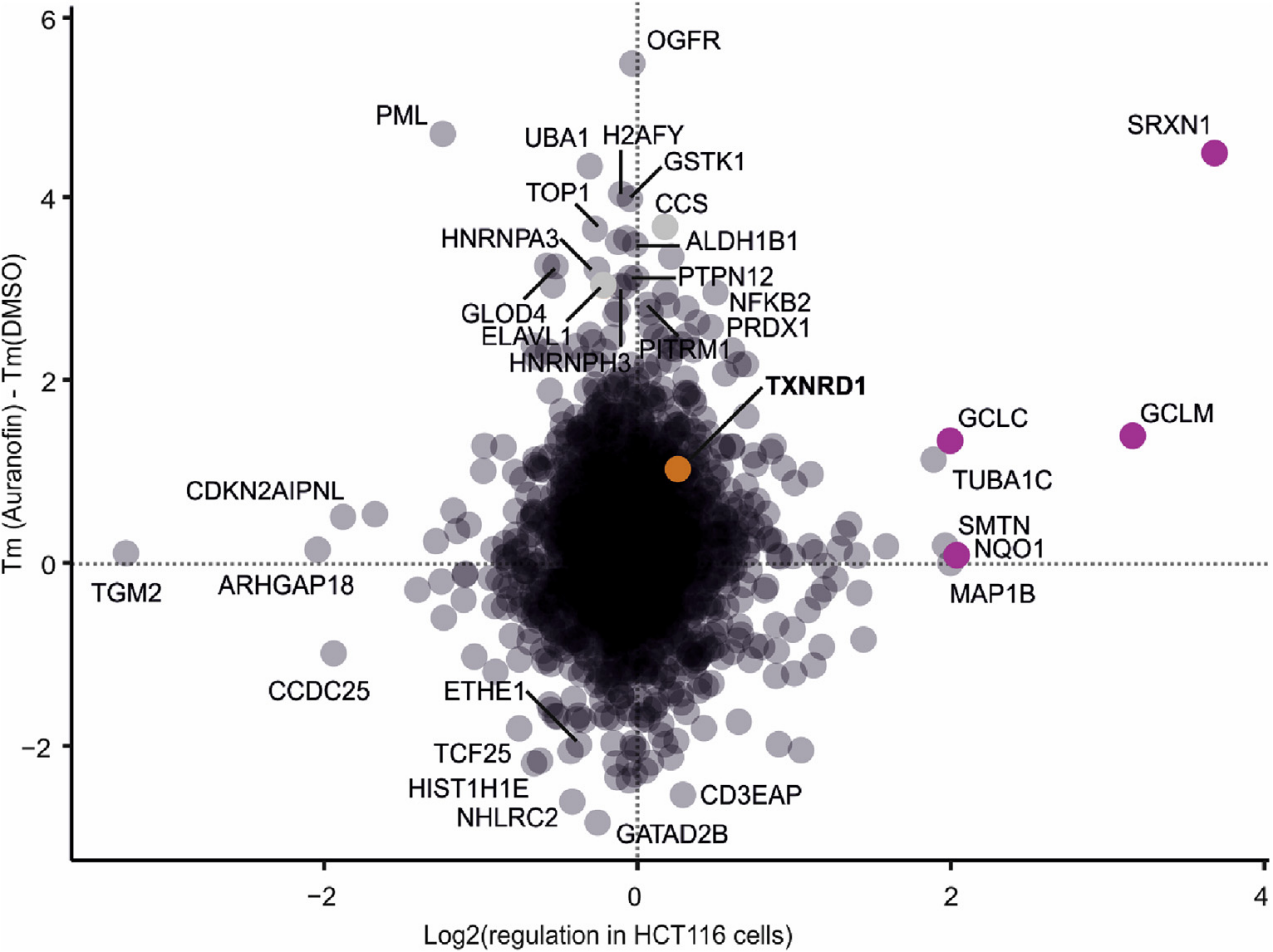
**Comparison of protein expression vs. thermal stability for HCT116 cells shows the orthogonality of FITExP and TPP**. Some Nrf2 target genes are shown in purple. (For interpretation of the references to color in this figure legend, the reader is referred to the Web version of this article.)

It is important to assess the complementarity and orthogonality of different chemical proteomics methods. Fig. 5 demonstrates that, in general, protein expression levels used in FITExP are orthogonal to thermal stability in TPP. Some proteins, such as SRXN1, GCLM and GCLC, are altered both in expression and stability upon auranofin treatment. The increased levels of these proteins might be to increased expression rates and/or secondary to stabilization of the proteins followed by increase protein half-lives. As mentioned before, some of these proteins are Nrf2 activated proteins. For example, SRXN1 which is one of the top-regulated proteins in FITExP, was also stabilized in TPP in cells. Other upregulated proteins with simultaneous expression changes and shifts in stability were GCLM and GCLC. OGFR is an example of a protein changing only in the stability dimension.

## 4 Conclusions

In summary, we here used three orthogonal chemical proteomics tools in analyses of the effects of the clinically used redox-active compound auranofin on cultured human cancer cells. We investigated how these techniques performed with respect to *de novo* deconvolution of drug targets and mechanisms of action. Our comprehensive proteomic signatures can be considered as resources in auranofin research, especially in light of its repurposing potential for cancer treatment. We demonstrated the complementarity of these techniques in identification of the cognate target TXNRD1 and we found NFKB2 and CHORDC1 as additional top targets. The pronounced effects on NFKB2 can in part be downstream of TXNRD1 targeting, with targeting of both proteins contributing to the chemical efficacy of auranofin. Less is known about the potential importance of CHORDC1, which should be studied further. Finally, we here showed that redox proteomics can be efficiently used for studies of downstream redox effects in correlation of protein stability changes with oxidation states of corresponding protein-derived peptides. We conclude that orthogonal chemical proteomics tools should be used in parallel for an increased confidence in identification of drug targets and cellular down-stream effects.

## Funding statement

This work was supported by a grant 2015.0063 from KAW to EA and RZ, and 2017–19 from VINNOVA to RZ. A.A.S. would like to acknowledge VINNOVA-funded Research School in Drug Discovery and Development (DDD) for funding his internship in Biochemical and Cellular Assay National facility, Science for Life Laboratory Drug Discovery, Stockholm, Sweden.

## Author contributions

P.I.A., K.J., E.S.J.A., and R.A.Z. conceived the project; A.A.S., H.G., B.L., K.J., P.I.A. and R.A.Z., designed the MS experiments; A.A.S., P.S., H.G. and B.L. performed the TPP experiments; A.A.S. and P.S. performed the FITExP, redox proteomics and other MS experiments; A.A.S. and C.M.B. did the data analysis; H.G., B.L., and P.I.A. contributed to the manuscript; A.A.S., E.S.J.A. and R.A.Z. wrote the manuscript.

## Declaration of competing interest

Katarina Johansson is currently an employee of Pfizer Innovations AB. The remaining authors declare no conflicts of interest.
