# Supplementary material for: Comprehensive chemical proteomics for target deconvolution of the redox active drug auranofin
Source: Redox Biol. 2020 Mar 3;32:101491. doi: 10.1016/j.redox.2020.101491 (PMC7082630; doi:10.1016/j.redox.2020.101491)
Supplement: Multimedia component 1 [file mmc1.docx]

**Supplementary Materials**

**Comprehensive chemical proteomics for target deconvolution of the redox active drug auranofin**

Amir Ata Saei, Hjalmar Gullberg, Pierre Sabatier, Christian M. Beusch, Katarina Johansson, Bo Lundgren, Per I. Arvidsson, Elias S.J. Arnér, Roman A. Zubarev

**Supplementary tables**

**Supplementary Table 1.** LC50s measured for the compounds in 48h treatment

|  | **HCT116 cells** | **A375 cells** | **RKO cells** |
| --- | --- | --- | --- |
| **Auranofin** | 1.5 µM | 1 µM | 250 nM |
| **Methotrexate** | 25 nM | 20 µM | 25 nM |
| **Paclitaxel** | 23 nM | 40 nM | 10 nM |

**Supplementary Table 2.** Details of the proteomic experiments performed in the current paper

| **Exp. No** | **Experiment** | **Cell line** | **Treatment** | **Duration of treatment** | **No. of samples** | **Quantification mode** | **Instrument** | **Total gradient time (min)** | **MS1 scan range (m/z)** | **HCD collision energy** | **Orbitrap resolution** | **MS/MS resolution** | **MS1 AGC target** | **MS2 AGC target** | **MS1 max injection time** | **MS2 max injection time** |
| --- | --- | --- | --- | --- | --- | --- | --- | --- | --- | --- | --- | --- | --- | --- | --- | --- |
| 1 | TR-TPP cells | HCT116 | 2 µM auranofin | 2h | 32 | TMT10 | Q Exactive Plus | 120 | 375-1400 | 35 | 70000 | 35000 | 3E6 | 2E5 | 250 | 120 |
| 2 | TR-TPP cells | HCT116 | 3 µM auranofin | 2h | 32 | TMT10 | Fusion | 180 | 400-1600 | 40 | 120000 | 60000 | 3E6 | 1E5 | 50 | 105 |
| 3 | TR-TPP lysate | HCT116 | 500 nM auranofin | 1h | 32 | TMT10 | Fusion | 140 | 400-1600 | 40 | 120000 | 60000 | 1E6 | 1E5 | 50 | 105 |
| 4 | FITExP | HCT116, A375 and RKO | 1.5 µM, 250 nM and 1 µM auranofin, respectively | 48h | 45 | Label-free | Fusion | 190 | 300-1500 | 30 | 120000 | 30000 | 4E5 | 1E5 | 50 | 60 |
| 5 | Redox experiment | HCT116 | 3 µM auranofin | 2h | 2 pooled | iodoTMT6 | Fusion | 240 | 400-1600 | 40 | 120000 | 30000 | 1E6 | 5E4 | 50 | 80 |
| 6 | Redox experiment fractionated | HCT116 | 3 µM auranofin | 2h | 16 | iodoTMT6 | Fusion | 130 | 400-1600 | 40 | 120000 | 30000 | 1E6 | 5E4 | 50 | 80 |

**Supplementary Table 3.** Curve fitting results for TPP in cells treated with 2 µm auranofin for 2 h (Excel)

**Supplementary Table 4.** Curve fitting results for TPP in cells treated with 3 µm auranofin for 2 h (Excel)

**Supplementary Table 5.** Curve fitting results for TPP in HCT116 cell lysate treated with 500 nM auranofin for 2 h (Excel)

**Supplementary Table 6.** The results of FITExP experiment (Excel)

**Supplementary Table 7.** Redox proteomics experiment in HCT116 cells treated with 3 µm auranofin for 2 h (Excel)

**Supplementary Table 8.** Redox proteomics experiment in HCT116 cells treated with 3 µm auranofin for 2 h (deep dataset after fractionation) (Excel)

**Supplementary Table 9.** Final ranking of 100 top proteins in all experiments (Excel)

**Supplementary Table 10.** Top target candidates ranked by various combinations of different chemical proteomics tools.

| **Ranking** | **TPP in cell and lysate, FITExP and redox proteomics** | **TPP in cells and lysate and FITExP** | **TPP in cells and lysate** | **FITExP** |
| --- | --- | --- | --- | --- |
| 1 | **NFKB2** | CCS | CCS | GCLM |
| 2 | **CHORDC1** | HPCAL1 | ELAVL1 | SQSTM1 |
| 3 | **TXNRD1** | LARP7 | RB1 | NQO1 |
| 4 | DNAJB1 | CTR9 | ALDH1B1 | GSR |
| 5 | GBE1 | **NFKB2** | ASH2L | GCLC |
| 6 | ECI2 | RTCA | WDHD1 | HSPA1A |
| 7 | HSP90AB1 | PRDX1 | ECI2 | PIR |
| 8 | PDIA3 | PITRM1 | HTATSF1 | SPCS3 |
| 9 | PDLIM7 | DNAJB1 | TRMU | G6PD |
| 10 | EXOSC5 | **CHORDC1** | **NFKB2** | PLP2 |
| 11 | PSMC1 | ALDH1B1 | DHFR | BLVRB |
| 12 | CSRP1 | **TXNRD1** | PITRM1 | **TXNRD1** |
| 13 | GEMIN5 | PKN2 | SSRP1 | BAG2 |
| 14 | NAA25 | WDR26 | DCTN2 | NAA25 |
| 15 | RAC1 | FABP5 | RAC1 | HIP1R |
| 84 | - | - | - | **CHORDC1** |
| 86 | - | - | **CHORDC1** | - |
| 116 | - | - | **TXNRD1** | - |
| 404 | - | - | - | **NFKB2** |
|  |  |  |  |  |

**Supplementary Table 11.** The enriched biological processes and molecular functions of 15 proteins from cluster 6 in **Fig. 3a** (from FITExP experiment)

|  | **#pathway ID** | **Pathway description** | **Number of proteins** | **False discovery rate** | **Matching proteins in the network (IDs)** |
| --- | --- | --- | --- | --- | --- |
| Biological Process (GO) | GO.0006749 | glutathione metabolic process | 4 | 0.000313 | G6PD,GCLC,GCLM,GSR |
|  | GO.0006979 | response to oxidative stress | 6 | 0.000478 | G6PD,GCLC,GCLM,GSR,NQO1,THBS1 |
|  | GO.0006805 | xenobiotic metabolic process | 4 | 0.0151 | GCLC,GCLM,NQO1,UGDH |
|  | GO.0043523 | regulation of neuron apoptotic process | 4 | 0.0151 | G6PD,GCLC,GCLM,NQO1 |
|  | GO.0051409 | response to nitrosative stress | 2 | 0.0151 | GCLC,GCLM |
|  | GO.0071466 | cellular response to xenobiotic stimulus | 4 | 0.0151 | GCLC,GCLM,NQO1,UGDH |
|  | GO.2001234 | negative regulation of apoptotic signaling pathway | 4 | 0.0151 | GCLC,GCLM,PLAUR,THBS1 |
|  | GO.0006534 | cysteine metabolic process | 2 | 0.0193 | GCLC,GCLM |
|  | GO.0006750 | glutathione biosynthetic process | 2 | 0.0392 | GCLC,GCLM |
|  | GO.0006915 | apoptotic process | 6 | 0.0392 | CTSL1,GCLC,GCLM,PLAUR,SQSTM1,THBS1 |
|  | GO.2001237 | negative regulation of extrinsic apoptotic signaling pathway | 3 | 0.0392 | GCLC,GCLM,THBS1 |
|  | GO.0051900 | regulation of mitochondrial depolarization | 2 | 0.0435 | GCLC,GCLM |
| Molecular Function (GO) | GO.0004357 | glutamate-cysteine ligase activity | 2 | 0.00206 | GCLC,GCLM |
|  | GO.0050662 | coenzyme binding | 4 | 0.0144 | G6PD,GCLC,GSR,UGDH |
| Cellular Component (GO) | GO.0017109 | glutamate-cysteine ligase complex | 2 | 0.000821 | GCLC,GCLM |
|  | GO.0005829 | cytosol | 10 | 0.00543 | BLVRB,FERMT1,G6PD,GCLC,GCLM,GSR,MAP1B,NQO1,SQSTM1,UGDH |
|  | GO.0070062 | extracellular exosome | 9 | 0.00649 | BLVRB,CTSL1,G6PD,GSR,NQO1,PLAUR,SQSTM1,THBS1,UGDH |
|  | GO.0044444 | cytoplasmic part | 12 | 0.0384 | BLVRB,CTSL1,FERMT1,G6PD,GCLC,GCLM,GSR,MAP1B,NQO1,PLAUR,THBS1,UGDH |
| KEGG | 480 | Glutathione metabolism | 4 | 1.12E-05 | G6PD,GCLC,GCLM,GSR |

**Supplementary Table 12.** The biological processes and molecular functions activated or upregulated in response to auranofin in the panel of cell lines (30 consistent top proteins from FITExP experiment)

|  | **#pathway ID** | **Pathway description** | **Number of proteins** | **False discovery rate** | **Matching proteins in the network (IDs)** |
| --- | --- | --- | --- | --- | --- |
| Biological Process (GO) | GO.0006749 | glutathione metabolic process | 4 | 0.00307 | G6PD,GCLC,GCLM,GSR |
|  | GO.0006979 | response to oxidative stress | 7 | 0.00307 | G6PD,GCLC,GCLM,GSR,NQO1,SRXN1,TXNRD1 |
|  | GO.0015820 | leucine transport | 2 | 0.0429 | SLC3A2,SLC7A5 |
|  | GO.0015827 | tryptophan transport | 2 | 0.0429 | SLC3A2,SLC7A5 |
|  | GO.0051409 | response to nitrosative stress | 2 | 0.0477 | GCLC,GCLM |
| Molecular Function (GO) | GO.0004357 | glutamate-cysteine ligase activity | 2 | 0.00492 | GCLC,GCLM |
|  | GO.0016209 | antioxidant activity | 4 | 0.00492 | GSR,NQO1,SRXN1,TXNRD1 |
|  | GO.0050662 | coenzyme binding | 5 | 0.00772 | AIFM2,G6PD,GCLC,GSR,TXNRD1 |
|  | GO.0016651 | oxidoreductase activity, acting on NAD(P)H | 4 | 0.0122 | AIFM2,GSR,NQO1,TXNRD1 |
|  | GO.0016491 | oxidoreductase activity | 7 | 0.0211 | AIFM2,BLVRB,G6PD,GSR,HTATIP2,PIR,SRXN1 |
|  | GO.0016668 | oxidoreductase activity, acting on a sulfur group of donors, NAD(P) as acceptor | 2 | 0.0437 | GSR,TXNRD1 |
| Cellular Component (GO) | GO.0005829 | cytosol | 16 | 0.00132 | AIFM2,BAG2,BLVRB,DNAJB4,EPB41L1,G6PD,GCLC,GCLM,GSR,KRT5,NQO1,SLC7A5,SQSTM1,SRXN1,STAM2,TXNRD1 |
|  | GO.0017109 | glutamate-cysteine ligase complex | 2 | 0.0017 | GCLC,GCLM |
|  | GO.0044444 | cytoplasmic part | 22 | 0.00339 | AGPAT9,AIFM2,BAG2,BLVRB,DNAJB4,EPB41L1,EPHX1,G6PD,GCLC,GCLM,GPC1,GSR,HIP1R,NAA25,NBAS,NQO1,PLP2,RSL24D1,SLC3A2,SLC7A5,SRXN1,STAM2 |
|  | GO.0005737 | cytoplasm | 24 | 0.0244 | AGPAT9,AIFM2,BAG2,BLVRB,DNAJB4,EPB41L1,EPHX1,G6PD,GCLC,GCLM,GPC1,GSR,HIP1R,HTATIP2,NAA25,NBAS,NQO1,PIR,PLP2,RSL24D1,SLC3A2,SLC7A5,SRXN1,STAM2 |
|  | GO.0044446 | intracellular organelle part | 21 | 0.0244 | AGPAT9,AIFM2,BLVRB,DNAJB4,EPHX1,G6PD,GPC1,GSR,HIP1R,HTATIP2,JUNB,KRT5,NAA25,NBAS,PLP2,RSL24D1,SLC3A2,SLC7A5,SQSTM1,STAM2,TXNRD1 |
|  | GO.0070062 | extracellular exosome | 12 | 0.0314 | BLVRB,DNAJB4,G6PD,GPC1,GSR,KRT5,NQO1,PLP2,SLC3A2,SLC7A5,SQSTM1,TXNRD1 |
|  | GO.0031988 | membrane-bounded vesicle | 13 | 0.0482 | BLVRB,DNAJB4,G6PD,GPC1,GSR,HIP1R,KRT5,NQO1,PLP2,SLC3A2,SLC7A5,SQSTM1,TXNRD1 |
| KEGG |  | Glutathione metabolism | 4 | 0.000218 | G6PD,GCLC,GCLM,GSR |

**Supplementary Table 13.** The biological processes and molecular functions down-regulated in response to auranofin in the panel of cell lines (30 consistent bottom proteins from FITExP experiment)

|  | **#pathway ID** | **Pathway description** | **Number of proteins** | **False discovery rate** | **Matching proteins in the network (IDs)** |
| --- | --- | --- | --- | --- | --- |
| Biological Process (GO) | GO:0016584 | nucleosome positioning | 3 | 0.00219 | CTCF,HIST1H1C,HIST1H1E |
| Molecular Function (GO) | GO:0031490 | chromatin DNA binding | 4 | 0.00578 | CTCF,H1F0,HIST1H1C,HIST1H1E |
| Cellular Component (GO) | GO:0000786 | nucleosome | 4 | 0.00719 | H1F0,HIST1H1C,HIST1H1E,HIST1H2AJ |
| PFAM Protein Domains | PF00538 | linker histone H1 and H5 family | 3 | 8.49e-05 | H1F0,HIST1H1C,HIST1H1E |
| INTERPRO Protein Domains and Features | IPR005819 | Histone H5 | 3 | 7.27e-05 | H1F0,HIST1H1C,HIST1H1E |
|  | IPR005818 | Linker histone H1/H5, domain H15 | 3 | 9.08e-05 | H1F0,HIST1H1C,HIST1H1E |

**Supplementary Table 14.** Peptides with altered oxidation and stability upon auranofin treatment

| **Gene name** | **Peptide sequence** | **Oxidation level ratio in auranofin vs. DMSO** | **Oxidation level ratio in DMSO vs. auranofin** | **P-value in redox experiment** | **Degree shift (auranofin –DMSO)** | **No of peptides in TPP** |
| --- | --- | --- | --- | --- | --- | --- |
| **PHF5A** | CVICGGPGVSDAYYCK | 2.50 | 0.40 | 0.00071 | +5.45 | 5 |
| **EREG** | CEVGYTGVR | 0.84 | 1.18 | 0.00296 | -1.60 | 3 |
| **TOP1** | AVAILCNHQR | 2.00 | 0.50 | 0.00645 | +3.65 | 10 |
| **TXNDC5** | IAEVDCTAER | 1.20 | 0.84 | 0.02127 | +2.48 | 28 |
| **TES** | CGQEEHDVLLSNEEDRK | 0.63 | 1.60 | 0.02462 | -1.49 | 25 |
| **PLRG1** | SPYLFSCGEDK | 1.92 | 0.52 | 0.02959 | +2.52 | 7 |
| **PRDX5** | GVLFGVPGAFTPGCSK | 0.59 | 1.70 | 0.03047 | -1.65 | 13 |
| **RRM1** | SAGGIGVAVSCIR | 3.00 | 0.33 | 0.03205 | +2.31 | 41 |
| **DDX5;DDX17** | STCIYGGAPK | 1.27 | 0.76 | 0.04153 | +1.27 | 39 |
| **SRXN1** | GAQGGDYFYSFGGCHR | 2.00 | 0.50 | 0.04309 | +4.48 | 6 |
| **MAP2K3** | ISCMSKPPAPNPTPPR | 1.56 | 0.64 | 0.04509 | +1.31 | 14 |
